# The Role of Fiber in Energy Balance

**DOI:** 10.1155/2019/4983657

**Published:** 2019-01-21

**Authors:** Astrid Kolderup Hervik, Birger Svihus

**Affiliations:** ^1^Inland Norway University of Applied Sciences and University of South-Eastern Norway, P.O. Box 4, 3199 Borre, Norway; ^2^Norwegian University of Life Sciences, P.O. Box 5003, 1432 Aas, Norway

## Abstract

Excessive energy intake is linked with obesity and subsequent diet-related health problems, and it is therefore a major nutritional challenge. Compared with the digestible carbohydrates starch and sugars, fiber has a low energy density and may have an attenuating effect on appetite. This narrative review attempts to clarify the net energy contributions of various fibers, and the effect of fiber on satiety and thus appetite regulation. Fibers, broadly defined as nonstarch polysaccharides, are a varied class of substances with vastly different physicochemical properties depending on their chemical arrangement. Thus, net energy content can vary from more than 10 kJ/g for soluble, nonviscous, and easily fermentable fibers such as those in many fruits, to less than zero for viscous fibers with anti-nutritive properties, such as certain types of fibers found in rye and other cereals. Likewise, some fibers will increase satiety by being viscous or contribute to large and/or swollen particles, which may facilitate mastication and increase retention time in the stomach, or potentially through fermentation and an ensuing satiety-inducing endocrine feedback from the colon. Thus, fibers may clearly contribute to energy balance. The metabolizable energy content is very often considerably lower than the commonly used level of 8 kJ per g fiber, and some fibers may reduce energy intake indirectly through satiety-inducing effects. A more precise characterization of fiber and its physicochemical effects are required before these beneficial effects can be fully exploited in human nutrition.

## 1. Introduction

Obesity is on the rise in affluent societies across the world [1]. Fundamentally, the nutritional cause of obesity is an excessive intake of carbohydrates and fat, which together contribute a majority of the dietary energy. Quantitatively, carbohydrates are the most important source of energy, but the different chemically defined classes of carbohydrates vary considerably in their contribution to energy intake. Starch is a polysaccharide composed of *α*-glucose linked through 1–4 and 1–6 bonds and is the quantitatively most important source of energy in the global diet [2]. The other important class of energy-providing carbohydrates in the diet is sugars, broadly defined as the monosaccharides glucose and fructose, and the disaccharides sucrose, maltose, and lactose [3]. Because of the complex contribution to sugar intake through added sugar, fruits, vegetables, and milk, accurate estimates are rare, but in the United States, sugars have been estimated to contribute to 23% of the energy intake in adults [4]. Similar data, based on calculations of fructose intake, have been found in Norway [5].

The remainder of the carbohydrates in the diet are the nondigestible oligosaccharides and the nonstarch polysaccharides. These carbohydrates are often considered beneficial in the diet because they cannot be broken down to monosaccharides which can be absorbed and used as an energy source. The nondigestible oligosaccharides are a varied group of low molecular weight saccharides containing more than two monosaccharide units. The main sources of these carbohydrates are fruits, vegetables, and legumes [6]. Generally, they are found in small quantities, although some foods such as Jerusalem artichokes and chicory may contain considerable quantities. Oligosaccharides are readily fermented by the gut microflora, and although small quantities may have beneficial gut health stimulating effects, consumption of large quantities may cause diarrhea [6].

The nonstarch polysaccharides, on the other hand, can be found in considerable quantities in many plant foods. They are a very diverse group of carbohydrates that can constitute a large part of the dry matter in many plant foods such as wheat bran and many vegetables. These carbohydrates are collectively included in the term fiber. Numerous review papers have focused on their potential role in contributing to better health, such as reducing the risk of obesity [7, 8], cardiovascular disease [9, 10], and diabetes [11, 12]. In terms of the number of persons affected, obesity is the most important dietary challenge in human nutrition. Obesity is also epidemiologically and causally strongly linked to cardiovascular disease and diabetes type 2. Understanding the mechanisms governing the influence of fiber on energy intake and thus obesity is therefore imperative. However, the exact physicochemical mechanisms governing the beneficial effect of fiber remain obscure, as well as the effect of different chemical constituents and sources of fiber. Thus, the current narrative review was carried out to cast light on this important issue, specifically focusing on the fundamental mechanisms governing the effect of fiber on energy balance in humans.

## 2. Materials and Methods

This narrative review is based on scientific peer-reviewed papers primarily obtained using a nonsystematic search of the databases Web of Science and PubMed. The initial step in the selection of literature was to identify relevant keywords to search for in these databases. Various combinations of the following keywords were used: “fiber/fibre,” “dietary fiber/fibre,” “fiber/fibre definition,” “physicochemical properties,” “energy value,” “fermentation,” “anti-nutritive effects,” “chemical analysis,” “satiety,” “satiation,” “appetite regulation,” “mastication,” “chewing,” “gastric retention time,” “gastric emptying rate,” “viscosity,” “particle size,” “transit time,” and “short chain fatty acids.” The search was conducted from April 2017 to April 2018. The subsequent step in the selection process was inclusion or exclusion of papers based on the relevance to the aim of the review. Both original and review papers were included. The reference lists of the included papers were also thoroughly studied to identify any possibly relevant papers. As far as possible, only original papers and reviews based on controlled trials and mechanistic studies were included. Unless otherwise specified, the results presented and discussed in this study are statistically significant. Animal studies have been included when found relevant, for example, because of lack of human studies.

## 3. Fiber Definition

Because of the complex nature and effects of fibers, the precise definition of fiber varies greatly. A chemically oriented definition, because of its preciseness, is a useful starting point. In its most simple definition, fibers are nonstarch polysaccharides [13]. In other words, saccharides composed of a large number of monosaccharides are linked through covalent bonds, which the human endogenous enzymes cannot break. However, this chemically sound and simple definition is of academic interest only because there are no viable analytical procedures which can be used to quantify this constituent of the diet precisely.

In practice, the term “dietary fiber” is commonly used and will be the basis for the definition of fiber here. In addition to the nonstarch polysaccharides, the term “dietary fiber” includes the lignin often associated with the fiber, which is not removed during the analytical procedure used to quantify dietary fiber [14]. Dietary fiber measured using this method will not include any starch because boiling with thermostable amylase removes all starch in the sample [15]. Starch has been defined as a fiber component if it is resistant to digestion in the small intestine, for example, due to chemical modification [16], and current dietary fiber analyses may include resistant starch in the analytical procedure [17]. The extent of indigestibility, however, varies considerably depending on the type of resistant starch and the method used for assessing digestibility [18]. The suitability of including this fraction among the fiber is therefore questionable. Resistant starch has also been discussed in several excellent reviews [18–20]. Thus, this carbohydrate component will not be considered a part of the fiber fraction in this review.

## 4. Physicochemical Properties of Fiber

### 4.1. Molecular Composition

Despite the common feature of not being digested by endogenous enzymes, fibers have vastly different physicochemical properties depending on their chemical arrangement. In addition to glucose, being the quantitatively most important building block of fiber because of its presence in cellulose and other nonstarch glucans, fibers may consist of or contain a number of other monosaccharides such as fructose, galactose, mannose, ribose, rhamnose, xylose, and arabinose. Of particular interest is the galacturonic acid found in pectins, which can produce strong gels in association with calcium ions [21]. The molecular size, the monosaccharide composition, the bonds involved (e.g., branching points) and the extent of lignification will determine the physicochemical properties of the fiber, and thus, the health effects.

### 4.2. Hydration and Viscosity

The exact nature of the interactions between physicochemical properties and physiological effects are not fully understood, but as pointed out by Bach Knudsen [14], hydration properties and viscosity effects are perhaps some of the most important. Hydration properties are interchangeably described as water-holding capacity and water-binding capacity, and this describes the ability of fiber to incorporate and hold water in its structure, which is measured as the amount of water a certain amount of fiber is able to hold. Insoluble fibers which are able to hold large quantities of water will often also swell. If the fibers are able to be dispersed in water, they are defined as soluble fibers. Soluble fibers are of nutritional importance because they may result in increased viscosity of the water and because soluble fibers are particularly easily fermented, as will be discussed below. The extent to which soluble fibers affect viscosity depends on the ability to form noncovalent bonds with surrounding water molecules and other fiber molecules. As will be discussed, viscous fibers may have specific nutritional effects through their potential satiating properties and their ability to interact with macronutrient digestion. As pointed out by Lovegrove et al. [22], both solubility and viscosity of fibers are complex and dynamic processes that are affected by numerous factors, and thus they are very difficult to estimate accurately. Amongst others, the viscosity of a fiber is determined by temperature, pH, fiber structure, chemical composition, molecular weight, and fiber concentration [13, 23–26]. Consequently, it has been demonstrated that the viscosity of foods is not necessarily transferable to the viscosity properties of the food in the gastrointestinal tract [27].

### 4.3. Particle Size

Particle size may also have important bearings on the physiological effects [28]. Encapsulation of nutrients in large particles, e.g., because of intact cell walls may be an impediment to digestion. In addition, as will be discussed below, large particles may slow down gastric emptying rate, thus increasing satiety. Encapsulation will affect nutrient availability and thus energy intake by physically impeding nutrients entrapped in the fibrous plant cell walls. This potentially important mechanism, because of its complexity and uncertain nature (e.g., particle size of foods, the effect of mastication, and physicochemical properties of the plant material), will not be dealt with here. Recent reviews give excellent overview of this topic [28, 29].

## 5. Energy Value of Fiber

### 5.1. Fermentability

Despite the fact that they are indigestible by human digestive enzymes, fibers may have an effect on energy value of foods in two opposing ways. Fibers will to varying extents be fermented by microflora in the colon, producing short-chain fatty acids (SCFA, mainly acetic, propionic, and butyric acid in a molar ratio of approximately 2.0 : 0.5 : 0.5), which subsequently will be absorbed and ultimately used as an energy source. A description of the microflora responsible for this effect is outside the scope of this review, but overviews have been published recently [30, 31]. Colonic enterocytes will oxidize most of the butyrate, while the liver will metabolize propionate, and the muscles and the brain will oxidize acetate [32]. It has been estimated that, in humans, 300 mmol of SCFA is produced per day, equivalent to 20 g of SCFA if a normal molar ratio of acetic, propionic, and butyric acid is assumed, and that a high capacity allows for a complete absorption in colon [33].

Obviously, the extent to which these SCFA are produced depends on the availability of substrates, chiefly carbohydrates, suitable for fermentation. However, the fermentability of different undigested carbohydrate sources also varies greatly. In vitro fermentation results reported by Cummings and Macfarlane [32] showed a yield of SCFA from as low as 10 g per 100 g for pea hulls, oat hulls, and cellulose to 40 g for pectins. This variability reflects both hydration and solubility properties of different fibers, both of which facilitate accessibility of fibers to fermentative bacteria. Because of the fact that fermentation of fiber by gut microflora results in energy loss, the net energy provided is lower than that inferred from proportion of fiber fermented, as will be discussed below.

### 5.2. Antinutritive Effects

Fibers may also reduce the energy value of foods through inhibiting digestion and absorption of other energy-providing macronutrients in the diet. Thus, Baer et al. [34] and Miles [35] found that both fat and protein digestibility was negatively affected when fiber content in diets increased. However, these results may have been confounded by other differences between the foods used in the different diets. A more valid approach would be to study the effect of fiber in isolation. Doing this, Castiglia-Delavaud et al. [36] found sugar beet fiber to result in a slight reduction (1%) in digestibility of fat but found no effect on nitrogen digestibility (corrected for microbial N). Farrell et al. [37] found a similar reduction in fat digestion and a significant reduction in digestibility of nitrogen when fibers in the form of bran were used as a fiber source. Wisker et al. [38] observed similar effects as Farrell et al. [37]. Wisker et al. [39] increased fiber content threefold by replacing refined wheat products with whole-grain wheat and rye and observed that nitrogen and fat digestibility was reduced from 87.4 to 79.6 and 96.4 to 93.6, respectively. Taneja et al. [40] observed increased fat excretion when 25 g/day of a viscous mucilaginous fiber was added to a standardized diet. However, others have found no effect of fiber, for example, when citrus fiber or barley fiber was used [41]. Nonetheless, taken together, fiber seems to be able to attenuate macronutrient digestibility.

The difference in response to fiber is probably at least partly due to physicochemical properties of fiber related to the ability of fiber to intervene in nutrient digestion and substrate absorption. Viscous fibers may be particularly effective, although the effects on nutrient digestibility have been mixed even with these types of fiber [25]. In a study by Ganji and Kies [42], subjects consumed standardized diets containing either soy oil or coconut oil (30% of the energy as fat) with and without 20 g of the highly viscous fiber psyllium. Quantitative collection revealed a reduction in fat digestibility of about 2 percentage units when psyllium fiber was consumed with the meal, with a significant increase in the amounts of palmitic and stearic acid excreted (transit time also decreased). In animal studies, the negative effect of viscous fibers on digestibility of nutrients is well documented, e.g., a reduction of ileal fat and protein digestibility in broiler chickens due to viscous (1–3) (1–4)beta-glucans from barley [43].

### 5.3. Quantifying Energy Value

In this section, an attempt will be made to quantify the energetic contribution of fiber, taking into consideration the effects mentioned above. Obviously, the energetic value of fiber will vary considerably as affected by fermentability and antinutritive effects. For example, Livesey [44] concluded in his review of energy value of fibers that the digestible energy value varied from −20 to +10 kJ/g. Thus, it is clear that the energetic value for fiber of 8 kJ/g as recommended by a FAO working group [45] is an average, at best.

The energetic value of different types of fiber has been assessed in a number of experiments. Wisker et al. [39], for example, carried out an experiment with a low-fiber and a high-fiber diet provided by cereal products. With the high-fiber diet, the subjects excreted more energy than the energy provided by the extra fiber, thus resulting in a negative energy value of the fiber component. This despite the fact that apparent digestibility of the fiber was 46.6 percent. The negative energetic value of the fiber was explained by an increased excretion of nitrogen and fat, thus indicating an antinutritive effect as discussed above. Similar results were found by Baer et al. [34]. In this experiment, subjects were fed diets with three levels of fat, where for each of these diets, the amount of fiber was varied to three levels by altering the amount of fiber from cereals and vegetables. For all the fat levels, a negative effect of increasing fiber on metabolizable energy content was found, indicating a negative energy value of the fiber. Apparent faecal digestibility of fiber was lower with high fiber content than with low fiber content but was rather high, varying between 67 and 82 percent. Fat and protein digestibility was significantly lowered with increasing fiber levels, particularly for diets with a high fat level, which can explain a negative energetic value of the fiber despite high digestibility.

Thus, as these data demonstrate, antinutritive effects may result in that fibers have a negative energetic value despite being partly degraded by the gut microflora. However, not all fibers have been shown to act like this. Wisker et al. [46], for example, found that the net energetic value of citrus fiber was 7.5 kJ/g when calculated based on energy lost in faeces, although this study also demonstrated that fiber from coarse rye bread had a negative net energy value of −2.1 kJ/g. Interestingly, all fiber sources had positive energetic value when calculated based on fermentability. In this method, which is based on an equation proposed by Livesey [44], net energy is calculated based on fermentability only and does not take into account antinutritive effects, which calculations based on energy loss in faeces will. Thus, the difference in net energy contribution of fiber from whole rye bread was 4.9 kJ/g when fermentation only was considered and −2.1 kJ/g when energy in faeces was taken into consideration; this demonstrates the large antinutritive effects for many fibers. Barley fiber also shifted from contributing positively to energy in the diet to contributing negatively when antinutritive effects were taken into consideration, while a small effect only was observed for citrus fiber.

Castiglia-Delavaud et al. [36] measured net energy content of isolated sugar beet fiber and inulin by the use of total collection of faeces and urine and estimation of heat loss through the use of respiration chambers. The latter is important because the net energy content of fibers is not only affected by fermentability and antinutritive effects but also by the heat produced and lost during digestion and metabolism. Thus, when energy is made available from a carbohydrate via fermentation rather than via enzymatic degradation to glucose, the heat loss will be larger. Net energy content of fibers will therefore be further reduced when heat loss is taken into consideration. Although the metabolizable energy content was rather similar at 10.7 and 13.0 kJ/g for both sugar beet fiber and inulin, the net energy value at 5 kJ/g for the former and 11.9 kJ/g for the latter demonstrated the large and variable effect of heat loss due to fermentation and metabolisation of energy from different fiber sources.

As the discussion above demonstrates, the energetic contribution of fiber varies considerably. The net energy value will sometimes be negative, and it will anyway usually not be higher than 8 kJ/g.

## 6. The Effects of Fiber on Satiety

### 6.1. Satiety Mechanisms

Intake of food will at some point reduce hunger and inhibit further food intake for a longer or shorter period. In this course of action, there are two processes involved: satiation and satiety. Satiation develops during an eating episode and causes meal termination, thus controlling meal size, whereas satiety occurs as a consequence of an eating episode and will temporarily inhibit further meal initiations [47–49]. Satiation is also known as intrameal satiety and satiety as intermeal satiety [49]. However, in studies of fiber and appetite this distinction is rarely used and thus the term satiety (or satiating effect) will be used in the following.

When discussing the effect of fiber on satiety, it is important to be aware of the complexity of the satiating process and factors affecting it. Clark and Slavin [50] point out that satiety and food intake may be influenced by many uncontrolled factors, such as stress level, environmental and social factors, palatability of the food, and sensory-specific satiety. Some consumers may, for example, find fiber-containing test meals unpalatable, an effect that may have impact on study results [51]. Blundell et al. [49] argue that the effect food has on satiety cannot be considered based on the effect of one component in isolation, as the effect may change when the component is eaten together with other foods. The effect of a particular fiber is thus depending on what this fiber is ingested together with [52]. In addition to the effect of different types of fibers as will be discussed below, there may also be differences within the same type of fiber, for example, due to differences in the way it is processed [53].

The effect of different types of fiber on satiety has been extensively reviewed. Gums [54], oat *β*-glucan [55], guar gum [56], pectin, alginate, and *β*-glucan [26] as well as fiber supplements and fiber-rich foods [57] have been concluded in reviews to have a satiating effect. On the contrary, others have concluded that most fiber types do not affect satiety [50] or that the effect of fiber on satiety is small [58]. Because fibers may have vastly different properties as discussed above, these diverging conclusions are not surprising. However, it seems clear that some fibers will have a satiating effect.

Fibers may affect satiety through several physiological mechanisms throughout the digestive tract. Here, the focus will mainly be on the effects of fibers on satiety through some physicochemical properties that seem to be especially linked to the means by which fibers can influence appetite regulation. These include physical structure effect, water-holding capacity, and viscosity. In addition, fermentable fibers will produce SCFAs in colon, which may also affect satiety. The satiating mechanisms will be explained, with a focus on how fibers via these physicochemical properties interact with the digestive mechanisms in the mouth, stomach, small intestine, and colon in ways that may impact satiety.

### 6.2. Oral Processing Time

Because fibers are part of the plant skeleton, they may be important for binding foods into large particles in what can be termed a physical structure effect. This property of fibers may increase oral processing time and effort needed for mastication [48, 59]. As indicated in a systematic review and meta-analysis [60], mastication seems to be linked to satiety. Wanders et al. [61] showed oral exposure time to be longer and food intake to be reduced when subjects ate a cookie containing alginate, but not guar gum or cellulose, compared with cookies without added fiber. A longer oral processing time seems to stimulate cephalic phase responses, i.e., responses to sensory signals that are sent out to prepare the digestive tract for the ingested material, and these are proposed to contribute to satiety [60, 62].

### 6.3. Gastric Volume and Retention Time

Ingestion of food will result in gastric distention, which via the vagus nerve will send signals to the brain leading to a feeling of fullness and satiety [62–64]. Gastric distention will also inhibit secretion of the hunger-stimulating hormone ghrelin [65]. The duration of gastric distention will be determined by gastric emptying rate, which again will be affected by the nature of food eaten [66]. Water together with food particles of small size move relatively fast through the stomach, whereas food particles of larger size with more mass will have longer retention time [29]. Fiber properties affecting these processes such as particle size, water-holding capacity, and viscosity are thus properties central to the effects different fibers may have on gastric retention time. In addition, effects of fibers in the lower gastrointestinal tract may also influence gastric-emptying rate via endocrine regulation [52].

In several human studies, fibers have been shown to delay gastric emptying [67–78]. Delay in gastric emptying may thus be one of the mechanisms explaining the satiating effect of fibers, and some properties of fibers seem to be especially important in this regard. When fiber enters the stomach, it will absorb water and swell. The amount of water absorbed and extent of swelling depends on the specific hydration properties of fibers [28]. The resulting increase in gastric volume will increase the gastric distension as mentioned above and hence lead to increased satiety. In studies of rats [79] and sows [80], where fiber increased gastric retention time, there are indications of this effect to be caused by the water-holding capacity of the ingested fiber. However, there seems to be a lack of human studies of the effect of water holding capacity of fiber on gastric retention time.

Increased viscosity of gastric load is another, and more well-documented, mechanism that may slow down gastric emptying and in this way facilitate satiety. Juvonen et al. [81] reported that gastric-emptying rate, measured by paracetamol absorption, was slower after intake of a high viscosity oat bran drink compared with a low-viscosity oat bran drink. This indicates an important role of viscosity in reducing gastric emptying rate. Also, Marciani et al. [73] showed that fiber-rich high-viscosity meals reduced gastric-emptying rate, gave greater gastric volumes and thus resulted in a higher satiety than low-viscosity meals.

In several studies, a reduced gastric-emptying rate has been reported after intake of viscous fibers such as guar gum [67], guar gum and pectin [68], pectin [69, 71, 75], *β*-glucan [72], and alginate (depending of dose) [76], although some studies have not demonstrated such effects [51, 61, 82–84]. The reduced gastric emptying rate discussed above may explain why some viscous fibers have been demonstrated to induce satiety [76, 85, 86], and that fiber-containing meals and drinks with high viscosity have been reported in several studies to increase satiety more than both low viscosity meals and drinks without [87, 88] and with fiber [51, 73, 89–91]. However, an oat bran beverage with low viscosity was demonstrated to increase satiety more than a high viscosity oat bran drink [81], thus demonstrating the complexity of these effects. Wanders et al. [58] concluded in their systematic review of randomized controlled trials that highly viscous fibers reduced appetite and food intake more than less viscous fibers. Even though scholars state that more clinical evidence is necessary [24, 66], it seems that viscous fibers have a potential to increase satiety.

The physical structure effect may impact gastric emptying rate [92]. In the stomach, it has been claimed that solid particles must be smaller than 1 to 2 mm in size in order to pass through pylorus and enter duodenum [93, 94]. Food particles of larger size will thus potentially need more time for size reduction than smaller particles before they will be allowed to enter duodenum, as has been demonstrated in animal studies [95, 96]. As mentioned above, fibers may contribute to larger particle size in foods and this may thus be one cause for the satiating effects of fibers. However, fiber-rich food sources vary greatly in particle size [97]. Thus, Vincent et al. [78] observed that intake of coarse bran, but not fine bran, prolonged gastric retention time in humans.

### 6.4. Small Intestinal Transit Time

Several authors have postulated that fibers have satiating effects through a prolonged small intestinal transit time [24, 26, 27, 55, 57]. Although bran has been shown to decrease small intestine transit time in humans [77, 98], there is a paucity of human studies where small intestinal transit time after intake of fiber has been studied. This potential effect of fiber therefore remains unsubstantiated.

### 6.5. Production of SCFAs in Colon

When entering colon, fibers will be fermented to various degrees. The fermentability of soluble fibers is generally much greater than that of insoluble fibers [99]. As discussed previously, the main products of fermentation of fiber in the colon are, together with gases, the SCFAs propionate, butyrate, and acetate [100]. The effect of these SCFAs in the gut is postulated to be of importance for appetite regulation [55]. This is due to the demonstration of SCFAs as ligands to receptors (free fatty acid receptors 2 and 3) present on the L-cells of the intestine [101–103]. These L-cells are endocrine cells known to produce peptide YY (PYY) and glucagon-like peptide-1(GLP-1), hormones that may reduce appetite and food intake [104–107]. In vitro studies have demonstrated that SCFAs stimulate PYY gene expression in rat gut cells [108], as well as release of PYY and GLP-1 in colonic cells from humans [109] and mice [110]. In animal studies, SCFAs have also been reported to stimulate secretion of GLP-1 and PYY [111], and it has been reported to result in reduced food intake [112].

Other mechanisms of SCFAs influencing satiety have also been suggested. SCFAs may affect motility in the upper gastrointestinal tract, which amongst others may reduce gastric emptying [113]. In mice, an appetite-reducing effect of acetate through interactions with the central nervous system has also been reported [114]. However, it is uncertain whether all these effects are transferable to the effects of SCFA in humans [99]. It has also been indicated that high doses of fibers are necessary for such effects in animals and humans [115, 116], and high fiber doses may cause negative side effects like bloating and flatulence. Because of the lack of well-controlled human interventions, the role of colon fermentation in human energy balance thus remains to be fully established [117, 118].

## 7. Conclusions

**Figure 1:**
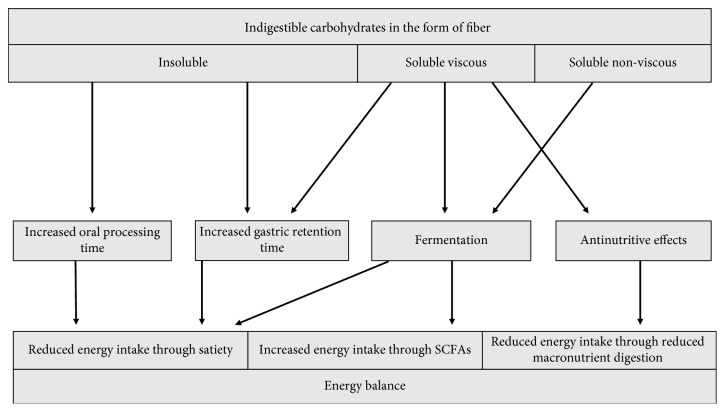
Potentially important mechanisms for fiber effect on energy balance. The size of boxes and arrows are arbitrary and are not indications of magnitude.

Two clear conclusions can be drawn from this overview. One is that fibers may clearly contribute to energy balance in an affluent society because of its very low and sometimes even negative energy value and because of satiety-inducing effects (Figure 1). The energetic value is related mainly to fermentability and antinutritive effects, where viscous soluble fibers may have a considerable net negative energy value due to attenuating effects on macronutrient digestion and absorption, whereas soluble nonviscous fibers will contribute moderately to energy through fermentation. Fibers may have effects on satiety at different stages of the digestive process and in varying parts of the digestive tract, depending on their physicochemical properties. It seems that some fibers may increase oral processing time, increase gastric retention time and possibly have effects in the gut via SCFA production that may contribute to satiety. Physical structure, hydration properties, viscosity, and fermentability are properties of fiber that may have impact on the capability of fiber to satiate via these processes.

However, the magnitude of these effects is difficult to assess, not the least because the nature of the fibers may affect energy contribution and satiety in opposing ways. This leads to the second clear conclusion of this review, namely that due to the extremely varied properties of fibers, broad general conclusions on the effect of fiber are difficult to make. Soluble and viscous fibers such as those found in barley and rye may be particularly beneficial for satiety through increased gastric retention and through fermentation in the colon, while simultaneously will often have a negative net energy value because of antinutritive effects. Other fiber components such as soluble but nonviscous fibers from, e.g., some fruits, may have a much lower or insignificant satiating effect due to less effect on gastric retention and will contribute to energy intake through a positive energy value as a consequence of lack of antinutritive effects and a rather complete fermentation in the colon.

Thus, until more research has been carried out to map the effects of different fiber types in regards to physiological digestive responses, only broad and careful general conclusions such as those above can be made. As this review demonstrates, fibers will contribute to energy through fermentation, albeit often much less than the value of 8 kJ/g as currently used in the calculation of energy content, especially when soluble, viscous fibers are considered, as these may have a negative net energy value. Fibers may also contribute to energy balance by having a satiating effect through increased oral processing time, gastric retention, and/or fermentation, although the magnitude of this effect is dependent on delicate and sometimes contradicting effects related to the physicochemical structure of the fiber.

### 7.1. Challenges and Future Directions

For a more precise description of the physiological effects of fiber, a more detailed distinction of the different types of fibers is needed, as also stated recently by others [119]. One possible option would be to distinguish between soluble and insoluble fibers because the solubility is an important prerequisite for rapid fermentation. Furthermore, soluble fibers could be classified into viscous and nonviscous types as an attempt to take potential antinutritive effects and satiating effects into consideration. Likewise, the insoluble fibers could potentially be classified according to their structural properties. This will take account of their potential diverging satiating properties depending on a number of still too poorly understood factors, e.g., their contribution to increased oral processing time and/or increased retention time because of particle size in the stomach. Given the complications in regard to the current fiber definition, such a detailed characterization of fibers will be challenging. After all, the current fiber definition where resistant starch is included have made fibers even more complex and varied in regard to physiological response, due to variable digestion of starch.

As this review demonstrates, more research is needed to clarify the effects of different fibers on energy balance and the effect on satiety in particular. Human studies using diets only differing in content of carefully characterized specific fibers would allow for a more precise use of fibers to obtain energy balance.
